# Cardiac Involvement in Systemic Lupus Erythematosus

**DOI:** 10.31083/RCM42760

**Published:** 2025-11-21

**Authors:** Vrinda Vyas, Vandita Vyas, Akash Sharma, Prashanth Ashok Kumar

**Affiliations:** ^1^Cardiology, ECU Health, Tarboro, NC 27886, USA; ^2^Anesthesiogy/Critical Care, Marienhospital, 70199 Stuttgart, Germany; ^3^Division of Cardiac Electrophysiology, The University of Kansas Medical Center, Kansas City, KS 66103, USA; ^4^Centre for Global Health Research, Saveetha Medical College and Hospital, Saveetha Institute of Medical and Technical Sciences, Saveetha University, 602105 Chennai, Tamil Nadu, India; ^5^Hematology-Oncology, George Washington University, Washington, D.C. 20037, USA

**Keywords:** cardiac, SLE, endocarditis, myocarditis, pericarditis

## Abstract

Systemic lupus erythematosus (SLE) is a chronic autoimmune disease caused by the production of autoantibodies, which form pathogenic immune complexes that deposit in multiple organs, leading to the multisystem involvement characteristic of SLE. Cardiovascular complications contribute substantially to the morbidity and mortality associated with SLE. Thus, this review discusses the cardiac manifestations of SLE, including the epidemiology, risk factors, pathogenesis, clinical features, diagnosis, and treatment options. Furthermore, we discuss the role of autoantibodies, endothelial dysfunction, and immune complex-mediated injury in the pathogenesis of SLE. Finally, we discuss emerging therapies and future research directions aimed at mitigating cardiac complications in SLE.

## 1. Introduction

Systemic lupus erythematosus (SLE) is a chronic autoimmune inflammatory disease affecting multiple organ systems, including the skin, serous membranes, joints, kidneys, heart, lungs, and nervous system [1]. The disease course is characterized by intermittent flare-ups interspersed with periods of remission. Some estimates suggest that SLE affects about 48–366.6 per 100,000 individuals [2] with a predilection for reproductive-age women; a female-to-male ratio of 13:1 [1]. The disease also exhibits a higher prevalence among individuals of African, Asian, and Hispanic ancestry [3, 4]. The leading causes of death in SLE were active disease flares and severe infections, with estimated 5-year mortality reported to be as high as 40% in the 1950s [5]. However, advances in immunosuppressive therapies and improvements in supportive care have shifted this trend. Now, cardiovascular (CV) disease has emerged as a primary cause of mortality in this patient population, with current 10-year survival rates estimated to be between 85 and 93% [6]. Cardiac involvement in SLE can involve all parts of the heart, including the pericardium, myocardium, valves, conduction system, and coronary arteries. Hence, CV manifestations encompass a broad spectrum of pathologies, including pericarditis, myocarditis, valvular abnormalities such as Libman–Sacks endocarditis, accelerated atherosclerosis, arrhythmias, and even heart failure (HF) [7]. While cardiac manifestations may occasionally precede the diagnosis of SLE, they more commonly develop insidiously in patients with established disease.

The underlying pathophysiology of cardiac involvement in SLE is multifactorial. In SLE, there is a breakdown in immunological self-tolerance, leading to the production of autoantibodies. These autoantibodies then react with “self-antigens” and form circulating immune complexes that deposit in various tissues, including the heart and blood vessels, thereby initiating further inflammation-endothelial dysfunction, complement activation, and cytokine-mediated cellular injury [8, 9]. In some cases, the production of antiphospholipid antibodies (APL) further complicates the clinical picture by causing thrombotic events.

This review aims to analyze the current literature on the cardiac manifestations of SLE. We will explore their epidemiology, underlying mechanisms, clinical features, and implications for diagnosis and management, with attention to recent advances and ongoing areas of investigation.

## 2. Epidemiology and Risk Factors 

Studies have shown that more than half of all individuals diagnosed with SLE will experience some form of cardiovascular complication over the course of their illness [7].

Pericarditis is the most common cardiac manifestation of SLE, while atherosclerotic diseases, including coronary artery disease (CAD), are significant causes of mortality. In a recent meta-analysis of studies, the relative risk of CV disease amongst SLE patients was estimated to be 2.35 [10]. The Framingham Offspring Study revealed that women aged 35–44 with SLE have a 50-fold higher risk of myocardial infarction (MI) compared to age-matched controls and a seven-fold higher risk of CAD after adjusting for other factors [11]. A study found the absolute 10-year MI risk in SLE patients to be 2.17% (95% CI: 1.66–2.80) compared to 1.49% (95% CI: 1.26–1.75) in healthy controls [12]. A cohort study of over 4000 SLE patients reported an MI incidence of 9.6 events per 1000 person-years (95% CI: 8.9–10.5), versus 4.9 events per 1000 person-years (95% CI: 4.8–5.1) in healthy subjects [13]. Additionally, CAD, angina pectoris, and MI were significantly more common in SLE patients compared to age-matched controls (15.2% vs. 3.6%, *p* = 0.0041) [14]. Mortality in SLE displays a bimodal distribution; early mortality is linked to organ inflammation and thrombosis, while later stages are often due to chronic inflammation and the development of atherosclerosis, leading to increased morbidity from CV diseases like CAD and HF [2, 15]. A meta-analysis highlighted that SLE patients experience 2.6 times higher mortality, primarily due to renal damage, CV disease, and infection [16, 17]. Various demographic and clinical factors influence the likelihood and severity of cardiac involvement in SLE. While SLE is more prevalent among women, male patients often experience a more severe disease course, including higher rates of CV complications and premature death [18, 19].

Additionally, individuals of African American and Hispanic descent tend to exhibit greater disease activity and poorer CV outcomes, potentially due to a combination of biological and socioeconomic factors [3, 4]. Age at onset and disease duration also contribute to cumulative CV risk over time [15]. Dyslipidemia caused by SLE characterized by low high-density lipoprotein (HDL) and high triglyceride levels, but notably, low-density lipoprotein (LDL) levels are not elevated, although oxidized LDL levels are increased [20, 21].

The accelerated vascular injury in SLE is hypothesized to stem from chronic systemic inflammation, immune complex deposition, and persistent endothelial dysfunction [22, 23, 24] this is further enhanced by the use of steroids. Additionally, lupus nephritis has been independently linked to a higher risk of CV disease, likely due to its role in promoting systemic inflammation and vascular damage [10].

## 3. Pathophysiology of SLE

CV involvement in SLE arises from a complex interplay of immunologic, inflammatory, genetic, environmental, and vascular factors that cause immune system dysregulation, chronic inflammation, endothelial dysfunction, vascular injury, and a predisposition for vascular thrombosis [8]. Together, these mechanisms contribute to a diverse range of multi-organ pathologies. A hallmark of SLE is the production of pathogenic autoantibodies—most notably anti-double-stranded DNA (anti-dsDNA), anti-Smith (anti-Sm), and APL antibodies. These autoantibodies target specific self-antigens, leading to the formation of circulating immune complexes. The immune complexes then get deposited within tissues and vascular beds, trigger activation of the complement cascade, and cause further downstream inflammatory activation [25]. Endothelial dysfunction represents a central pathogenic mechanism in SLE. While increased expression of adhesion molecules such as vascular cell adhesion molecule-1 (VCAM-1) and intercellular adhesion molecule-1 (ICAM-1) facilitates leukocyte recruitment to the vessel wall, the abnormalities extend beyond simple up-regulation. SLE patients demonstrate disturbed shear-stress responses, with impaired mechanotransduction and altered wall shear stress—known to regulate endothelial nitric oxide synthase (eNOS) and vascular tone—contributing to endothelial injury and atherosclerosis [26, 27]. In addition, there is a pronounced angiogenic imbalance in SLE: elevated angiopoietin-2 and reduced angiopoietin-1 serum levels reflect dysregulated tyrosine kinase with immunoglobulin-like and ECF-like domains 2 (Tie2) signaling, impairing vascular stability and angiogenesis, and these changes are driven in part by type I interferons and correlate with disease activity [28]. These disruptions are compounded by microcirculatory disturbances specific to SLE—nailfold capillaroscopic studies consistently reveal capillary rarefaction, dilated and tortuous loops, and reduced capillary density, all of which correlate with disease activity and organ involvement [29].

The production of APL antibodies, such as lupus anticoagulant, anticardiolipin, and anti-β2 glycoprotein I antibodies, causes vascular thrombosis in SLE. These antibodies have been shown to cause endothelial and platelet activation, complement-mediated injury, and disrupt the normal fibrinolytic processes, thereby leading to both arterial and venous thromboembolic phenomena and valvular injury [30, 31]. The presence of these thrombophilic autoantibodies has been linked to increased rates of CAD, cerebrovascular accidents, and pregnancy-related complications [32].

Together, these mechanisms perpetuate inflammation and thrombosis, which contributes to accelerated atherosclerosis and other CV manifestations seen in SLE. In the following sections, we explore how these pathogenic processes give rise to the specific clinical cardiac manifestations of SLE, including pericarditis, myocarditis, valvular heart disease, accelerated atherosclerosis, arrhythmias, and HF.

## 4. Pericarditis

Pericarditis is the most frequent cardiac manifestation of SLE and was also the first cardiac complication recognized as secondary to the disease. Asymptomatic pericarditis is believed to be even more common than clinically apparent cases. Estimates suggest that about one in four patients with SLE experience pericarditis during the course of their illness [33]. Autopsy studies have reported pericardial involvement in 60–80% of cases [6, 33]. Most patients have subclinical pericardial inflammation; however, when clinical pericarditis occurs, it typically presents with retrosternal chest pain that worsens with inspiration or improves when sitting forward. Other symptoms may include fever, tachycardia, and dyspnea. When associated with pericardial effusion, physical findings can include muffled heart sounds, a pericardial friction rub, or pericardial knocks. Although rare, pericarditis can occasionally be the first manifestation of SLE [34]. A large multicenter study of ~1400 patients reported that pericardial effusion, detected by echocardiography, occurs in 10–50% of lupus patients [35]. Cardiac tamponade, though uncommon, has been reported in up to 15% of cases, with varying prevalence across studies [36]. Although rare, some patients may even develop recurrent pericarditis or effusive-constrictive pericarditis as a result of chronic inflammation of the pericardial lining. Immune complex deposition has been thought to be the pathophysiologic mechanism behind lupus pericarditis. Direct immunofluorescence staining has shown the deposition of complement factors and immunoglobulin on the pericardium.

Those with the presence of anti-Jo-1, anti-DNA, and anti-Smith antibodies have been seen to be at an increased risk of developing lupus pericarditis [37, 38]. Genomic investigations have found specific single nucleotide polymorphisms which may predispose SLE patients to pericarditis [39]. Pericarditis may cause changes in the electrocardiogram (EKG), including PR segment depressions and diffuse concave upward ST segment elevations. Echocardiography, while not routinely recommended in all SLE patients, may be beneficial in those cases where pericarditis is suspected. Computed tomography (CT) imaging showing pericardial thickening or effusion may also be beneficial. Especially in instances of recurrent pericarditis, an accurate diagnosis may require modalities such as cardiac magnetic resonance (CMR) and CT.

The treatment of pericarditis varies based on severity; while most patients can be treated with nonsteroidal anti-inflammatory drugs alone, some patients may need corticosteroids and even intravenous immunoglobulin for refractory or recurrent cases. Colchicine can be safely used in lupus pericarditis [40]. Other immunosuppressants such as methotrexate, mycophenolate, azathioprine, and anakinra may be used to effectively manage underlying lupus flare-ups. Pericardiocentesis may be required in some circumstances [41]. Pericardial fluid in SLE is usually exudative, with elevated leukocytes, a pH <7, and sometimes the presence of autoantibodies.

## 5. Myocarditis

Myocarditis, while occurring less frequently than pericarditis, has been estimated to occur in about 40–70% of SLE patients in an autopsy study [42]. Myocarditis accompanying SLE portends a more severe clinical condition than pericarditis due to its possible impact on heart function, with a mortality rate of around 10% [43]. Clinically symptomatic myocarditis has been estimated to occur in about 3–9% of SLE patients [44].

Clinical symptoms include chest pain, fever, dyspnea, palpitations, development of acute HF, cardiogenic shock, heart blocks, and ventricular arrhythmia in severe cases [44]. Early identification and treatment of SLE myocarditis is essential for preventing progression and initiating timely treatment. Findings include elevated Erythrocyte sedimentation rate (ESR) and troponins [42]. EKGs may usually indicate nonspecific findings such as sinus tachycardia; however, ventricular arrhythmias, ST-T abnormalities, and conduction blocks can also be seen [45]. Echocardiography may demonstrate global hypokinesia, wall motion abnormalities, and decreased left ventricular ejection fraction [43, 46]. Chest X-ray can be completely normal or may indicate cardiomegaly or pulmonary vascular congestion, depending on the severity of SLE myocarditis. Cardiac MRI using T2-images may demonstrate delayed gadolinium enhancement or a pattern or mid myocardial and subepicardial enhancement, which are nonspecific to SLE but may be suggestive of SLE-related myocarditis in patients with known SLE diagnosis [43, 47]. The more invasive endomyocardial biopsy remains the gold standard for diagnosis of lupus myocarditis. Patients with known SLE and newly decreased cardiac function should first undergo ischemia evaluation, and once CAD has been excluded, an endomyocardial biopsy should be performed to exclude alternative etiologies such as viral myocarditis [48].

Biopsy findings are nonspecific and could be similar to other types of immune-mediated myocarditis, with the presence of perivascular and interstitial infiltrate composed of leukocytes with areas of collagen degeneration, myocyte necrosis, and interstitial edema and thickening of vessel walls with fine granular immune complex deposition [49].

There is no clear consensus regarding the best treatment for SLE-related myocarditis, as there have not been many large-scale randomized clinical trials. However, intravenous (IV) corticosteroids and rituximab therapy have been shown to be beneficial [50, 51]. One study demonstrated that aggressive immunosuppressive therapy for SLE reduced the odds of lupus myocarditis occurring [46]. ESC guidelines recommend confirming the diagnosis with endomyocardial biopsy to exclude infectious causes before immunosuppression. High-dose IV methylprednisolone pulses followed by an oral taper, often with azathioprine, cyclophosphamide, or intravenous immunoglobulin (IVIg), are advised [52]. SLE myocarditis can lead to life-threatening arrhythmias, HF, conduction abnormalities, and even sudden cardiac death.

## 6. Endocarditis/Valvular Heart Disease

The reported frequency of endocardial and valvular involvement in SLE varies significantly.

Valvular involvement varies from 11% to 25% in different studies [53, 54] this increases to 13%–75% in autopsy [7]. In a meta-analysis, SLE tends to have an 11-fold increased risk of combined valvular alterations, and a 10-fold increase in mitral valve or aortic valve thickening, mitral valve regurgitation, and mitral valve vegetation when compared to controls without SLE [55].

The classic cardiac valvular abnormality seen in SLE patients is called Libman-Sacks endocarditis (LSE) or marantic or nonbacterial thrombotic endocarditis, and as the name suggests, it involves the thickening and development of noninfective/sterile verrucous vegetation on the cardiac valves [56]. Prevalence of LSE is 1 in 10 SLE patients [45]. It primarily affects the mitral valve, followed by the aortic valve, although multivalvular abnormality may also be seen [57, 58]. Valvular regurgitation, valve stenosis, chordal rupture, intracardiac thrombosis, and systemic thromboembolic events have all been reported as complications of valvular involvement in SLE [7].

The vegetations are red-tan, granular, verrucous lesions ranging from 1–12 mm, usually located near valve edges, commissures, chordae tendineae, or adjacent endocardium [7, 59]. In LSE, the vegetations are composed of endothelial cells, platelets, fibrin, and fibroblasts and may even have enlarged macrophages called anitschkow cells, which are usually found within granulomas [60]. Vegetations may demonstrate evidence of immunoglobulin and immune complex deposits and the presence of hematoxylin bodies on histopathological analysis [7, 59]. Healed vegetations are characterized by microscopic examination demonstrating fibrosis without active inflammation.

While LSE itself rarely causes valve perforation—helping differentiate it from bacterial infective endocarditis (IE)—its presence predisposes patients to secondary IE. Antiphospholipid antibodies (APLs) play an important role in valvular pathology. Multiple studies and meta-analyses have shown a strong association between APL positivity and valvular disease [61]. In one meta-analysis of 23 studies (n = 1656), valvular disease was present in 43% of APL-positive patients versus 22% of APL-negative patients (OR = 3.13, *p* < 0.00001) [62]. Retrospective data further suggest an association between LSE and β2-glycoprotein and lupus anticoagulant antibodies [63] as well as anti-cardiolipin antibody positivity [64]. Longer duration and more severe SLE, the presence of APL antibodies, anticardiolipin, or lupus anticoagulant antibodies has been shown to be associated with risk for LSE development [53]. These antibodies most likely have a role in the development of pathogenic immune complexes in SLE patients, but their exact function in the pathogenesis of valvular involvement in SLE needs to be further studied. However, it may be suggested that SLE patients with APL, anticardiolipin, LA or B2 glycoprotein antibodies may benefit from routine echocardiography screening.

Similar to other types of cardiac involvement in SLE, the majority of the patients may be asymptomatic, but around 1–2% may require surgery [65]. Those who do develop symptoms may present with dyspnea or signs and symptoms of left-sided HF, mostly from valvular regurgitation or stenoses [60].

A high degree of clinical suspicion is needed to make the diagnosis of LSE. In patients with suspected valvular involvement, transthoracic and/or transesophageal echocardiogram may be necessary. Blood cultures may be required to exclude infective endocarditis. A hypercoagulable workup including for lupus anticoagulant and antiphospholipid antibodies levels may be required as well. In certain cases, cancer may need to be excluded as the appearance of nonbacterial thrombotic endocarditis from cancer is indistinguishable from that of SLE.

The optimal treatment for LSE is not well understood, and recommendations vary. Anticoagulation should be considered for secondary prevention for thromboembolic phenomena in patients who have had a prior thromboembolic event, but it is not necessarily recommended for patients with LSE alone. Warfarin with an INR goal of 2 to 3 has been considered the gold standard in patients with antiphospholipid syndrome secondary to SLE. In cases of significant valvular dysfunction, surgery should be considered [41]. As per European society of cardiology 2023 endocarditis guidelines, because thromboembolic events are a major risk in Libman–Sacks endocarditis, anticoagulation is frequently recommended to minimize this risk. In cases where significant valvular dysfunction develops, such as severe mitral regurgitation, interventional strategies may be necessary. Surgical repair or replacement has been traditionally used, but less invasive approaches, such as transcatheter edge-to-edge repair (TEER), have also shown success in carefully selected patients [66].

## 7. Accelerated Atherosclerosis/Ischemic Heart Disease

Coronary artery involvement in SLE can be secondary to atherosclerosis, arteritis, thrombosis, and vasospasm. Atherosclerotic CV disease represents a major cause of late morbidity and mortality in SLE patients. Studies have shown that SLE patients have an increased risk of CAD, fatal, and even nonfatal MI [67, 68].

In a study comparing the Hopkins Lupus Cohort with the Framingham controls, there was seen to be a 2.66-fold increased risk of CV disease [69]. The incidence of MI in SLE patients is estimated to be 9-to-50-fold higher compared to the general population [48]. Fatal MI has been reported to be 3 times higher in patients with SLE than in age- and sex-matched control subjects [70, 71]. Although not all SLE patients develop symptoms, autopsy studies reveal significant coronary atherosclerosis in 25%–50% of patients, irrespective of cause of death. One study using thallium exercise stress testing demonstrated perfusion defects consistent with CAD in 38% of SLE patients [7]. Surprisingly, the risk of MI in young women with SLE has been reported to be up to 50-fold higher than in the general population [11]. As young women do not usually have traditional CAD risk factors, accelerated atherosclerosis is suspected in SLE [22]. The exact pathogenesis of premature atherosclerosis in SLE remains unclear. Proposed mechanisms include immune complex deposition, leading to vessel wall inflammation and vasculitis [23]. In SLE, proinflammatory cytokines such as tumor necrosis factor-α (TNF-α) and IL-1 are released, which then recruit leukocytes to the vessel endothelium. These cells then propagate further inflammation by increasing vascular expression of adhesion molecules, ultimately leading to endothelial dysfunction and vasculitis [72].

Also, this inflammatory milieu causes circulating LDL to get oxidized, leading to foam cell formation and, ultimately, atherosclerotic plaque formation within the vessel media [73]. Elevated levels of homocysteine and Lipoprotein (a) (Lp(a)) are also noted in SLE patients [74]. Together, chronic inflammation, endothelial dysfunction, and oxidative stress along with steroids use are considered central to premature atherosclerosis in SLE. The role of autoantibodies is also significant. Antiphospholipid and anti-endothelial cell antibodies are associated with an increased risk of CV disease, although mechanisms remain unclear [75]. Other antibodies, including anti-dsDNA, anti-Ro, and anti-Sm, have also been linked to CV risk [76].

The symptoms, diagnosis, and management of coronary atherosclerosis in patients with SLE are the same as those in patients without SLE. Single photon emission computed tomography stress test, coronary artery calcium score, and coronary artery CT scan may be useful in SLE patients with suspected atherosclerotic CVD [77, 78].

Some trials have studied carotid ultrasound to detect subclinical atherosclerosis in SLE patients.

One trial was conducted in pediatric patients with SLE, studying the benefit of statin in preventing subclinical atherosclerosis progression. The trial did not show any significant effect of routine statin use in preventing progression of carotid intimal-media thickening as measured by carotid ultrasonography [79].

Another study showed a similar lack of benefit of statin in reducing subclinical atherosclerosis in adult SLE patients [80]. The Lupus Atherosclerosis Prevention Study (LAPS) found that in a 2-year randomized trial of 200 SLE patients without overt CVD, atorvastatin 40 mg daily did not significantly slow subclinical atherosclerosis progression or improve SLE activity or inflammatory markers [80]. However, post-hoc analyses, such as from the pediatric APPLE trial, suggest potential benefit in patients with high baseline inflammation. Observational studies, including a large Taiwanese cohort, indicate statins may reduce mortality and CV events in SLE patients with dyslipidemia, particularly with high-intensity therapy [81]. Early randomized controlled trials (RCTs) in largely unselected SLE cohorts (e.g., LAPS, atherosclerosis prevention in pediatric lupus erythematosus trial (APPLE)) were neutral on surrogate atherosclerosis endpoints, but contemporary guidance and newer cohort data support statin therapy in SLE when atherosclerosis is present or ASCVD risk is clearly elevated. The 2024 EULAR SLE management recommendations emphasize treating dyslipidemia per general-population standards and recognize SLE as a risk-enhancing condition—thereby endorsing statins for patients with established ASCVD or imaging-confirmed plaque/CAC [82]. Hydroxychloroquine has also been hypothesized to be beneficial due to its lipid-lowering and antithrombotic properties in CAD patients with lupus [83]. After atherosclerosis, arteritis is the next most common type of coronary artery involvement in patients with SLE. Coronary artery aneurysms or rapidly developing stenoses or occlusions on serial angiograms are not pathognomonic but support a diagnosis of arteritis. Clinically, the distinction is important because the treatment of arthritis is to increase the corticosteroid dose, whereas a higher corticosteroid dose can worsen risk factors and potentially prove to be detrimental to a patient with atherosclerotic disease. Warfarin and antiplatelet agents have been used in patients with arteritis in an attempt to reduce the risk of thrombotic occlusion.

## 8. Cardiac Arrhythmias

Cardiac arrhythmias are increasingly being recognized as features of SLE-related cardiac disease. While sinus tachycardia is the most commonly associated, other reported cardiac arrhythmias in SLE patients include atrial fibrillation, sinus tachycardia, atrial tachycardia, and ventricular arrhythmias.

In one study of 235 SLE patients who underwent electrocardiography, sinus tachycardia was found in 18%, sinus bradycardia in 14%, QT prolongation in 17%, and tachyarrhythmias (including atrial fibrillation (AF), atrial flutter, atrial tachycardia, and atrioventricular nodal reentrant tachycardia/atrioventricular reentry tachycardia [AVNRT/AVRT]) in 6% of patients [84]. Another study found that patients with SLE develop atrial fibrillation more frequently than the general population [6]. Barnado *et al*. [85] confirmed the independent association between SLE and AF, after adjusting for age, sex, race, and CAD. Furthermore, SLE patients were noted to have almost twice the risk of hospitalization due to AF when compared to those without SLE [86]. Other conduction abnormalities, including atrioventricular blocks, intraventricular conduction delays, and sick sinus syndrome, have also been reported. The prevalence of sinus node dysfunction in SLE patients was reported as 4.3% with 3.6% requiring pacemaker implantation over a 5-year follow-up [87]. Long QT syndrome may occur, especially related to hydroxychloroquine toxicity has also been seen [88, 89, 90]. However, the findings of another study found no correlation between whole blood hydroxychloroquine levels and QTc intervals in patients with SLE treated with hydroxychloroquine [91].

These arrhythmias have been postulated to result from myocarditis, cardiac ischemia, or immune-mediated injury to the conduction system. Holter monitoring is valuable for detecting intermittent arrhythmias in SLE patients with suspected cardiac complications. Treatment is based on the type of arrhythmia/conduction disturbance. Benign tachyarrhythmias may be managed conservatively with beta-blockers or calcium channel blockers, alongside control of underlying SLE-related systemic inflammation. Corticosteroids may be indicated in arrhythmias related to active myocarditis. Further research is necessary to understand the specific inflammatory pathways responsible for the correlation between SLE and various arrhythmias.

Neonatal lupus occurs due to maternal anti-SSA/anti-Ro antibodies crossing the placenta and attacking antigens within the fetal conduction tissue, leading to congenital heart block (CHB) [92]. Congenital heart block is recognized in utero or post-delivery in neonates born to mothers with SLE. It occurs in an estimated 1 in 17,000–22,000 births in the general population and carries a 9–25% risk of fetal death [93].

In anti-SSA/Ro-positive mothers who previously had a child affected with CHB, the risk of recurrence remains high, and the fetus should be monitored closely with fetal echocardiography every week at 16–26 weeks of pregnancy. Though some therapies have been used to prevent CHB, there is no data from large randomized trials. Potential therapies include steroids, intravenous immunoglobulin, and hydroxychloroquine. Corticosteroids like dexamethasone, which can cross the placenta have been shown to be beneficial and prevent progression of fetal conduction issues to CHB [94, 95]. One estimate suggests up to 64% of children born with CHB will require pacemaker placement in the first year of life. A retrospective review of 15 infants born with CHB found that all had pacemaker insertion. One died, but the overall outcome was determined to be favorable [96, 97].

## 9. Heart Failure

Heart failure is recently being recognized as a potential complication of SLE. It is thought to result from a variety of etiologies and can be a consequence of myocarditis, severe valvular regurgitation or stenosis, CAD, or chronic hypertension/hypertensive heart disease.

The clinical prevalence varies in different studies, though the true incidence is likely underestimated due to subclinical presentations in most patients. Patients with SLE have up to a twofold higher risk of developing HF compared to the healthy people and the coexistence of SLE and HF contributes to increased mortality [8]. HF in SLE may manifest as systolic dysfunction from myocarditis or ischemic heart disease, or as diastolic dysfunction due to fibrosis, hypertension, or valvular disease. Endothelial inflammation, microvascular dysfunction, and long-term corticosteroid use may further contribute [98].

One retrospective cohort study demonstrated HF incidence was markedly higher in SLE patients compared to controls (0.97% (950/95,400) vs 0.22% (97,950/45,189,140), relative risk (RR): 4.6 (95% CI 4.3 to 4.9)). It also showed that the RR of HF was highest in young males with SLE (65.2 (35.3 to 120.5) for ages 20–24). That study also showed that renal involvement in SLE correlated with earlier and higher incidence of HF [99]. Symptoms and signs of HF in SLE are similar to those in non-SLE populations, including exertional dyspnea, orthopnea, edema, and fatigue. Echocardiography is the primary diagnostic modality, supported by elevated brain natriuretic peptide (BNP) or NT-proBNP levels. Cardiac MRI (CMR) may be used to assess myocardial inflammation or fibrosis and to rule out other potential HF etiologies. In a study, a high prevalence of subclinical HF in SLE patients was confirmed with CMR imaging [100]. Diagnosis of HF in SLE patients does not differ from those for the general population, and include comprehensive analysis of clinical signs and symptoms, laboratory and echocardiographic assessments. While the role of GDMT is unclear in SLE-related HF as it has not been studied in randomized trials, treatment usually incorporates standard HF, with the addition of immunosuppressive therapy when active inflammation is present. Wu *et al*. [101] developed a prognostic tool based on CMR imaging to identify SLE patients at a higher risk of developing HF. This tool should be validated in future studies and indicates towards a future screening with imaging to identify SLE patients at risk for CVD [101].

Monitoring for renal function and drug-related cardiotoxicity is crucial. Prognosis depends on the underlying cause and response to therapy, but outcomes can be significantly improved with early diagnosis and management [102]. Non-pharmacologic strategies play an important role in comprehensive care. These include individualized exercise regimens adjusted for disease activity, heart-healthy dietary modifications, psychosocial support, and a strong emphasis on medication adherence. Patient education is central to empowering individuals with SLE to participate actively in their long-term care, thereby improving clinical outcomes.

## 10. Future Directions

Despite advances in disease-modifying therapies, CV complications remain a leading cause of morbidity and mortality in SLE. High lupus activity was shown to be associated with increased CV risk, so lupus activity should be controlled in SLE patients [69]. Future research efforts must prioritize earlier detection, more precise risk stratification, and personalized therapeutic approaches to mitigate the burden of cardiac disease in this population. One area of active investigation involves the identification and validation of novel biomarkers for early cardiac involvement. High-sensitivity cardiac troponins, natriuretic peptides, anti-endothelial cell antibodies, and circulating microRNAs, all of which have shown preliminary utility in small studies, need to be further studied. Advancements in imaging technologies, such as CMR, PET-CT, offer enhanced sensitivity in detecting subclinical myocardial inflammation and fibrosis. One recent study demonstrated that patients with late gadolinium enhancement on CMR are more likely to develop CAD, myocarditis, pericarditis, HF, and atrial tachycardia, suggesting that CMR should be considered as part of routine screening and prognostication in SLE patients. CMR can also be beneficial in identifying the underlying pathophysiology of myocardial injury [103].

However, currently there are no specific recommendations for the use of CMR in SLE, probably due to poor availability and cost issues. Another promising area of future research involves the use of artificial intelligence in imaging which may have prognostic value in patients with normal CMR/imaging [104, 105]. Table 1 (Ref. [106, 107, 108]) represents the different imaging modalities with sensitivity and specificity.

**Table 1.** **Different imaging modalities used to diagnose myocarditis in SLE**.

Modality	Method/Criteria	Sensitivity	Specificity	Comments
Transthoracic echo (conventional)	LV/RV function, WMA, pericardial effusion	—	—	Routine TTE has low sensitivity for myocarditis and may be normal unless disease is moderate–severe. In SLE, routine echo is specifically noted to be low sensitivity for wall-motion abnormalities.
Speckle-tracking echo (STE) [106]	Global longitudinal strain (GLS_endo cut-off ≈ −21.35%)	71.9%	62.2%	In SLE, STE detects subclinical myocardial injury and is more sensitive than conventional echo, including when EF is preserved.
CMR (2009 Lake Louise) [107]	≥2 of: T2 oedema, EGE hyperaemia, non-ischaemic LGE	78–80%	87–88%	Widely validated in suspected myocarditis; strong tissue characterization. In SLE, CMR is recommended and often positive despite subclinical presentation.
CMR (2018 Revised Lake Louise) [108]	T2-based and T1-based markers (± mapping)	87.5%	96.2%	Revised criteria improve sensitivity while maintaining high specificity versus 2009 LLC; preferred when available.

LV, left ventricle; RV, right ventricle; WMA, wall motion abnormality; TTE, transthoracic echocardiography; SLE, systemic lupus erythematosus; STE, speckle-tracking echocardiography; GLS, global longitudinal strain; EF, ejection fraction; CMR, cardiac magnetic resonance; LLC, Lake Louise Criteria; T2, T2-weighted imaging (oedema marker); EGE, early gadolinium enhancement (hyperaemia marker); LGE, late gadolinium enhancement (non-ischaemic scar marker).

The therapeutic landscape is also evolving. Belimumab, a BAFF inhibitor approved for SLE, may offer CV benefits by improving HDL functionality, as shown in a study where 6 months of treatment enhanced cholesterol efflux, antioxidant activity, and paraoxonase-1 levels while reducing oxidized HDL lipids [109]. These effects, along with BAFF’s link to subclinical atherosclerosis, suggest potential vascular protection. Belimumab also lowers SLE activity and enables steroid sparing, indirectly reducing CVD risk by mitigating steroid-induced hypertension, diabetes, and hyperlipidemia [110]. Similarly, anifrolumab, a type I interferon receptor blocker approved in 2021, allows significant steroid reduction while controlling disease, addressing a major modifiable CVD risk factor in lupus. Type I interferon blockade may also directly reduce endothelial dysfunction and plaque formation, with the ongoing IFN-CVD trial evaluating vascular outcomes [111]. JAK inhibitors, such as tofacitinib and upadacitinib, target inflammatory pathways relevant to both lupus and atherosclerosis, with preclinical studies showing improved endothelial function and reduced vascular stiffness. TYK2 and JAK1 inhibitors are in phase III SLE trials, which may clarify their CV impact [112]. Other emerging agents, including IL-6, IL-1β, and BTK inhibitors, aim to reduce inflammation-driven CVD, echoing findings from non-lupus trials like CANTOS. Hydroxychloroquine remains a cornerstone, improving survival and lowering thrombotic risk through immune, metabolic, and antithrombotic effects. Together, these therapies represent a shift toward controlling SLE activity while targeting vascular pathology. If long-term data confirm CV benefit, they could reshape CVD prevention strategies in lupus.

Prevention of cardiac disease in SLE requires a multifaceted approach for both traditional cardiovascular risk factors and disease-specific mechanisms of injury. Rigorous control of systemic inflammation through the use of hydroxychloroquine, judicious glucocorticoid use, and timely initiation of immunosuppressive or biologic therapies is essential to reduce endothelial dysfunction and immune-mediated vascular damage. Standard preventive strategies—including aggressive management of hypertension, diabetes, and dyslipidemia; smoking cessation; weight control; and regular physical activity—should be implemented early, as SLE patients face a markedly elevated risk of premature atherosclerosis and MI. Statin therapy may be considered in those with persistent dyslipidemia or subclinical atherosclerosis. Antiplatelet or anticoagulant therapy should be used in patients with antiphospholipid antibodies who are at high risk for thrombotic events. Regular cardiovascular screening, including blood pressure monitoring, lipid profiling, and in select cases non-invasive imaging, further supports early detection and risk modification.

## 11. Conclusion

In conclusion, cardiac involvement in SLE is common, multifactorial, and a significant contributor to morbidity and mortality. Early recognition and comprehensive management of pericarditis, myocarditis, valvular disease, atherosclerosis, and arrhythmias are essential to improve outcomes. Advances in imaging, biomarkers, and targeted immunotherapies offer promise in mitigating CV risk in SLE. A multidisciplinary approach remains pivotal in delivering personalized, evidence-based care to this complex patient population.
